# Impact of Acute Hemoglobin Falls in Heart Failure Patients: A Population Study

**DOI:** 10.3390/jcm9061869

**Published:** 2020-06-15

**Authors:** Cristina Lopez, Jose Luis Holgado, Antonio Fernandez, Inmaculada Sauri, Ruth Uso, Jose Luis Trillo, Sara Vela, Carlos Bea, Julio Nuñez, Ana Ferrer, Javier Gamez, Adrian Ruiz, Josep Redon

**Affiliations:** 1 Cardiovascular and Renal Research Group INCLIVA Research Institute University of Valencia, 46010 Valencia, Spain; crislozu@incliva.es (C.L.); jlholgado@incliva.es (J.L.H.); tonifdz@yahoo.es (A.F.); isauri@incliva.es (I.S.); uso_rut@gva.es (R.U.); trillo.jlu@outlook.com (J.L.T.); ana.ferrer-albero@uv.es (A.F.); jgamez@incliva.es (J.G.); 2 Internal Medicine Hospital Clínico de Valencia, 46010 Valencia, Spain; sara.vela.b@gmail.com (S.V.); CARLOS.BEA@outlook.com (C.B.); anruhe@gmail.com (A.R.); 3 Cardiology Hospital Clínico de Valencia, 46010 Valencia, Spain; yulnunez@gmail.com; 4 CIBERObn Carlos III Institute Madrid, 28029 Madrid, Spain

**Keywords:** heart failure, anemia, blood loss, acute kidney injury, hospitalization, mortality

## Abstract

**Aims:** This study assessed the impact of acute hemoglobin (Hb) falls in heart failure (HF) patients. **Methods:** HF patients with repeated Hb values over time were included. Falls in Hb greater than 30% were considered to represent an acute episode of anemia and the risk of hospitalization and all-cause mortality after the first episode was assessed. **Results:** In total, 45,437 HF patients (54.9% female, mean age 74.3 years) during a follow-up average of 2.9 years were analyzed. A total of 2892 (6.4%) patients had one episode of Hb falls, 139 (0.3%) had more than one episode, and 342 (0.8%) had concomitant acute kidney injury (AKI). Acute heart failure occurred in 4673 (10.3%) patients, representing 3.6/100 HF patients/year. The risk of hospitalization increased with one episode (Hazard Ratio = 1.30, 95% confidence interval (CI) 1.19–1.43), two or more episodes (HR = 1.59, 95% CI 1.14–2.23, and concurrent AKI (HR = 1.61, 95% CI 1.27–2.03). A total of 10,490 patients have died, representing 8.1/100 HF patients/year. The risk of mortality was HR = 2.20 (95% CI 2.06–2.35) for one episode, HR = 3.14 (95% CI 2.48–3.97) for two or more episodes, and HR = 3.20 (95% CI 2.73–3.75) with AKI. In the two or more episodes and AKI groups, Hb levels at the baseline were significantly lower (10.2–11.4 g/dL) than in the no episodes group (12.8 g/dL), and a higher and significant mortality in these subgroups was observed. **Conclusions:** Hb falls in heart failure patients identified those with a worse prognosis requiring a more careful evaluation and follow-up.

## 1. Introduction

The relevance of anemia in the history and prognosis of heart failure (HF) has been recognized [1,2,3]. Well-established as a prognostic factor, the prevalence is more than 25% in all patients with HF and even greater in older adults [4]. Moreover, a progressive reduction of hemoglobin (Hb) implies a poor prognosis [5,6]. The origin of anemia in HF is multifactorial and includes fluid retention, chronic kidney disease, undernutrition, relatively low levels of erythropoietin, inflammation, and drugs blocking the renin-angiotensin system that reduce erythropoietin, erythroid progenitors, and the degradation of an inhibitor of erythropoiesis [3,7]. In addition, blood loss can contribute to significant reductions in Hb levels. Antiplatelet and anticoagulant treatment, with or without gastrointestinal lesions [8], as well as acute kidney injury (AKI), induce episodes of rapid Hb reduction [9]. The Hb fall not only impacts the comorbidities frequently present in HF patients, but also increases the myocardial workload [10].

Whilst the prevalence, mechanisms, and consequences of anemia in HF have been extensively studied, the impact of episodes with a significant decrease in Hb values has not been analyzed. Employing data from Electronic Health Records (EHRs), the prognostic value of acute anemia episodes in the risk of hospital admissions and mortality of HF patients is presented in this paper.

## 2. Subjects and Methods

### 2.1. Study Population and Baseline Data Collection

Patient data were collected from the EHRs of the Valencian Health Agency’s ABUCASIS, with a population of 3,799,885 subjects older than 18 years. From the original source, data on demographics, diseases, vital status, laboratory, and medication were transferred pseudo-anonymized to an external database, where a second process of anonymization was performed. The process of obtaining information fulfilled the Spanish Law of Data Protection of 3/2018 and the EU General Data Protection Regulation (GDPR) [11]. The Ethics and Clinical Trials Committee of the Valencia Hospital Clinic approved the study.

Among all of the subjects included in the database, a diagnosis of HF was recorded in 132,065 patients (International Classification of Disease (ICD-9) codes which identified HF patients in the EHR: 398.91, 402.01, 402.11, 402.91, 404.01, 404.11, 404.91, 404.03, 404.13, 404.93, and all 428). In the present study, we selected patients with data on Hb values repeated over time from 1 January 2012 until 31 December 2016. A total of 45,437 patients of both sexes fulfilled the eligibility criteria. During the study period, patients received treatment as part of the usual clinical practice.

### 2.2. Acute Anemia and Kidney Injury Assessment

Episodes of Hb falls greater than 30% of the baseline value with posterior recovery were identified. Hb falls produced as a consequence of blood collection due to medical or chirurgical procedures was excluded. The baseline Hb was defined as the weighted average of values first determined by performing linear interpolation between measurements, including data from both the baseline and one year prior to the study period. The number of Hb falls episodes, as well as the magnitude of the reduction, was recorded.

To assess renal function, the estimated glomerular filtration rate (eGFR) was calculated with the Chronic Kidney Disease Epidemiology Collaboration equation (CKD-EPI) [12] in parallel to the Hb values. A kinetic rate Jaffé method was used to measure the serum creatinine (sCr) (Boehringer Mannheim Diagnostics). Kidney Disease Improving Outcomes (KDIGO) stratification of chronic renal disease [13] was applied at the beginning of the observation period in stable conditions. A sudden increase of sCr with posterior recovery defined episodes of AKI [14]. The Risk, Injury, Failure, Loss of kidney function (RIFLE) scale [15] was used to estimate AKI severity, and a potential cause of Hb falls was those with injury (sCr 2-fold increase) or failure (sCr 3-fold increase or sCr >4.0 mg/dL) categories.

### 2.3. Cardiovascular Risk Factors Definition

The cardiovascular risk factors definition was previously published [16]. The values of blood pressure presented are the average of the blood pressure (BP) values registered. Hypertension was defined following the European Society of Cardiology / European Society of Hypertension ESC-ESH Guidelines [17] or a physician diagnosis in the record. Diabetes was defined when non-fasting glucose ≥200 mg/dL, serum HbA1c ≥6.5%, physician diagnosis in the record, or the use of insulin-lowering or oral glucose-lowering drugs was recorded. Serum cholesterol was measured by the cholesterol high performance reagent (Roche Diagnostics, Roche, Basel, Switzerland). A direct cholesterol high-density lipoprotein (HDL) reagent (Roche Diagnostics) was used and cholesterol low-density lipoprotein (LDL) was calculated by Friedwald’s formula. Total cholesterol >200 mg/dL and/or lipid-lowering drug treatment defined dyslipidemia.

### 2.4. Mortality and Hospitalization

From 1 January 2012 to 31 December 2016, Acute Heart Failure (AHF) hospitalization and all-cause mortality were recorded. Hospital records of HF as the main cause for admission and the Spanish National Death Index corroborated the status of each patient. The time to event was calculated for each of the outcomes—hospitalization and mortality.

### 2.5. Statistical Analysis

The Hb at the baseline and before the event or at the last recording was analyzed. Differences between the quantitative variables among groups were assessed using ANOVA with Bonferroni correction and qualitative variables by Chi-squared test. The relative risk (RR) for the incidence of Hb falls episodes was estimated by logistic regression analysis. To estimate the impact of Hb falls in the incidence of hospitalization by AHF or mortality, a Cox proportional hazard regression model was used. Potential confounding factors, consisting of age, sex, hypertension, diabetes, angiotensin-converting enzyme inhibitors (ACEi), angiotensin II receptor blockers (ARBs), antialdosterone, diuretics, and antiplatelet and oral anticoagulant drugs, were included in a multivariate Cox regression hazard model.

## 3. Results

### 3.1. General Characteristics of the Study Population

The general characteristics of the study population are shown in Table 1. Heart failure patients, with a total number of 45,437 individuals (54.9% female, mean age 74.3 year), were included. The number of subjects with one episode was 2892 (6.4%), two or more episodes was 139 (0.3%), and concomitant AKI was 342 (0.7%). A total of 3512 episodes of Hb falls in the 3373 patients (7.4%) was recorded during 2.9 years of follow-up, indicating an incidence of 2.8/100 HF patients/year. The episodes were more frequent in men and no differences in age were observed. The baseline glomerular filtration rate (GFR) was significantly lower in subjects with episodes mainly in the group in which decreased Hb levels occurred with AKI. Although there were significant differences in the baseline Hb among the groups, the values at the time of the event (hospitalization or mortality) were not different to those at the baseline. In addition, the average reduction of Hb during the episodes was 4.92 ± 1.2 g/dL. Among the comorbidities, baseline anemia, previous myocardial infarction, and atrial fibrillation were more frequent in patients with episodes. Treatments at the baseline are detailed in Table 1. More frequent use of diuretics, beta-blockers, antialdosterone drugs, non-steroidal anti-inflammatory drugs, antiplatelets, and oral anticoagulants was observed in patients with episodes compared to those without. Anticoagulant use, but not antiplatelet use, increased the risk of Hb fall episodes, with values of RR = 2.33 (95% confidence interval (CI) 2.17–2.51) and RR = 0.75 (95% CI 0.71–0.79), respectively.

### 3.2. Episodes of Hb Falls and Acute Heart Failure Hospitalization

**Figure 1:**
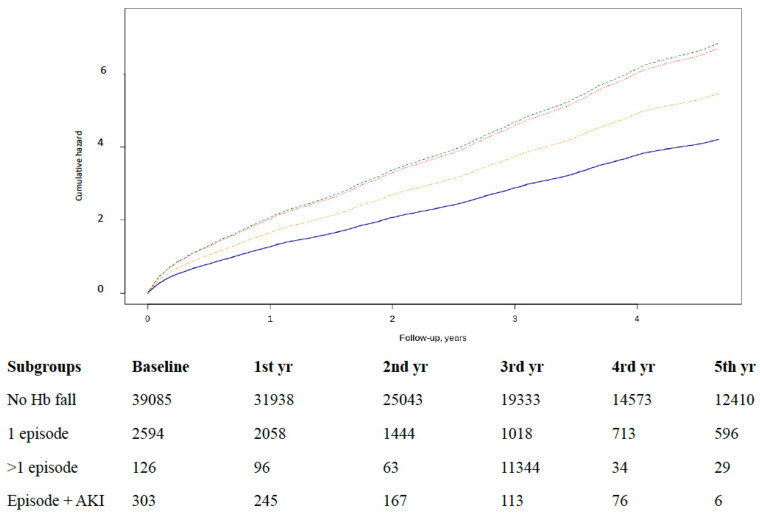
Cumulative risk of hospital admissions due to Acute Heart Failure by subgroup of patients with episodes of hemoglobin (Hb) fall with significant differences. No Hb fall (blue line): reference; one episode (yellow): HR = 1.30 (95% confidence interval (CI) 1.19–1.43); two or more (orange): HR = 1.59 (95% CI 1.14–2.23); and concomitant acute kidney injury (AKI) (green): HR = 1.61 (95% CI 1.27–2.03). Table number of subjects during the study.

Acute HF admission was recorded in 4673 (10.3%) patients, representing an incidence of 3.6/100 HF patients/year. Overall, 4260 (9.4%) patients displayed the absence of a previous episode of Hb fall, 348 (0.7%) patients had one episode, 23 (0.1%) patients had two or more episodes, and 42 (0.1%) also had AKI. Compared with the group without a drop in Hb levels, the risk of hospitalization significantly increased with episodes adjusted by age, sex, hypertension, diabetes, ACEi/ARB, diuretics, and anticoagulant and antiplatelet drugs. The increase of risk was observed in all patients with episodes: one episode: HR = 1.30 (95% CI 1.19–1.43); two or more: HR = 1.59 (95% CI 1.14–2.23); and concomitant AKI: HR = 1.61 (95% CI 1.27–2.03) (Figure 1). Furthermore, while the baseline Hb value reduced the risk, the magnitude of Hb reduction during the episodes did not increase the risk.

### 3.3. Episodes of Hb Falls and All-Cause Mortality

**Figure 2:**
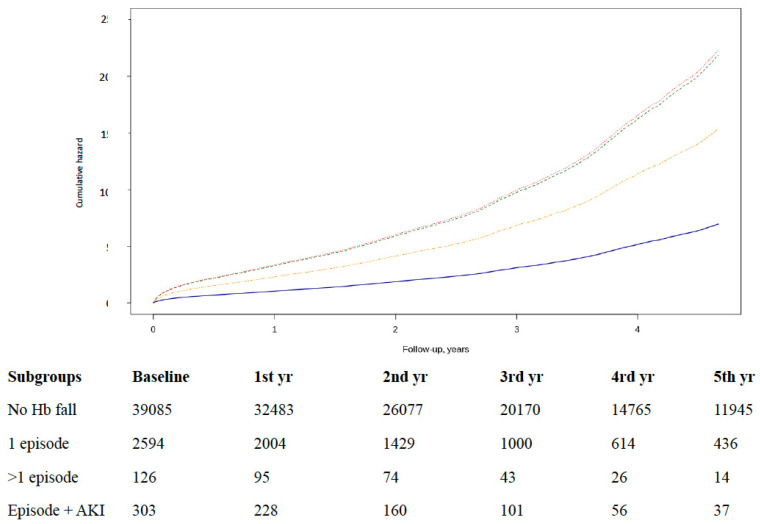
Risk of all-cause mortality by subgroup of patients with episodes of Hb falls with significant differences. No Hb (blue line): reference; one episode (yellow): HR = 2.20 (95% CI 2.06–2.35); two or more episodes (orange): HR = 3.14 (95% CI 2.48–3.97); and concomitant AKI (green): HR = 3.20 (95% CI 2.73–3.75). Table number of subjects during the study.

The number of deaths recorded was 10,490, indicating an incidence of 7.4/100 HF patients/year. Among them, 9037 (19.9%) had Hb fall episodes, 1191 (2.6%) had one episode, 83 (0.1%) had two or more episodes, and 179 (0.3%) had concomitant AKI. Compared with the group without Hb falls, the all-cause mortality significantly increased in patients with episodes adjusted by age, sex, hypertension, diabetes, ACEi/ARB, diuretics, and anticoagulant and antiplatelet drugs. The increase of risk was observed in all patients with episodes: one: HR = 2.20 (95% CI 2.06–2.35); two or more: HR = 3.14 (95% CI 2.48–3.97); and concomitant AKI: HR = 3.20 (95% CI 2.73–3.75) (Figure 2). Furthermore, the baseline Hb value reduced the risk, but the extent of Hb falls did not increase it.

## 4. Discussion

In a large cohort of HF patients, the impacts of significant Hb fall episodes on hospital admission due to AHF and all-cause mortality were analyzed. Episodes of Hb falls increased the risk of AHF hospitalization and all-cause mortality. Furthermore, the Hb reduction during the episodes did not seem to be relevant.

The present real-world data study includes a large cohort of HF patients in usual care, selected from a public general-practice covering 92% of the population living in the area. Details on the EHRs were previously published [16]. Information on the baseline risk factors, mortality, and hospitalization during follow-up was collected. To avoid potential bias in the Hb fall estimation secondary to the use of diuretics, ACEi, or congestion, we selected those with a reduction in Hb values greater than 30%. The baseline of Hb was calculated as the weighted average of values obtained from one year prior to the study period and the baseline. A simultaneous significant decrease in GFR was also recorded and analyzed as a subgroup, since this represents a cause of Hb falls [18] due to a combination of a greater risk of blood loss, platelet dysfunction, and reduced capacity to increase red blood cell production due to lower levels of erythropoietin [19].

The potential causes of Hb falls in these patients can be numerous and combine treatment with anticoagulants or antiplatelet drugs and frequent gastrointestinal lesions. Gastrointestinal reflux, duodenal ulcers, and angiodysplasia occur in more than 30% of this aged population [8,20]. In addition, patients with HF are at risk of developing AKI [21], due to a low cardiac output or congestive status, as well as the use of drugs blocking the renin-angiotensin system or diuretics. In an AKI condition, Hb falls is an accompanying element [21], mainly in the most severe classes, and increase the burden to the heart [18]. Whether the incidence of hospitalization or mortality was higher in patients with a reduced left ventricular ejection fraction than in those with a preserved ejection fraction was not possible to assess in the present study, due to the low number of left ventricular functions recorded in the EHR.

The impact of comorbidities on the prognosis of HF has been assessed in multiple studies that have tried to identify factors related to hospitalization and prognosis [22,23,24]. Concomitant ischemic heart disease, arrhythmias, renal dysfunction, non-compliance to treatments or diet, infections, uncontrolled hypertension, and anemia are relevant risk factors. The impact of one of the comorbidities, Hb fall episodes, on hospital admission by AHF or mortality was approached in the present study. It is well-known that anemia is a negative predictive factor for HF patients, both with a reduced [25] or preserved [26] ejection fraction. Likewise, anemia reduces the exercise capacity of HF patients and has been linked to more elevated levels of NT-proBNP and hs-troponin [27]. It is expected that the impact of Hb falls produces a similar effect, which is at least transitory during deep anemia episodes.

Concerning hospital admissions, we avoided counting the total number of hospitalizations since HF patients are admitted for other comorbidities and only those with AHF were considered. Episodes of significant Hb falls contribute to the fragility of patients, resulting in an increase in the future risk of hospitalization and mortality. It is worth noting that the baseline Hb value reduces the risk, despite the Hb falls; however, the extent of Hb falls in the episodes did not result in a higher risk. While the importance of the baseline Hb value reducing the risk is expected, the lack of impact of the amount of Hb fall during the episodes is less clear. If those with a larger Hb drop received blood transfusion, reducing the myocardial damage could be a potential explanation.

The strengths and limitations of the study should be considered. In the absence of criteria for defining significant Hb falls, we chose a 30% fall, considering that this relevant reduction cannot be produced by changes in blood volume due to congestion. All-cause mortality and no cardiovascular risk were assessed, since a precise identification of the cause was not possible. Likewise, no information about natriuretic peptides or troponin values and the left ventricular ejection fraction was available. The large number of HFs, however, allowed us to observe the impact of the episodes of Hb falls in terms of the prognosis in a real setting.

In conclusion, Hb falls produce an increase in risk beyond an acute episode in HF patients. With the progressive increase in the incidence of HF and hospitalization due to AHF, strategies to reduce the risk should include a more careful follow-up in patients with episodes of Hb falls.

## Figures and Tables

**Table 1:** General characteristics of the study population.

	All Subjects	No Hb Falls	One Episode Hb Fall	Two or More Hb Falls	AKI and Hb Falls
**Number**	45,437	42,064	2892	139	342
**Sex (M)**	20,504 (45.1)	18,568 (44.1)	1647 (57) *	91 (65.5) *	198 (57.9) *
**Body mass index (kg/m^2^)**	31 ± 5.7	31.1 ± 5.7	30.4 ± 5.5 *	29.4 ± 5.6	30.1 ± 5.4
**Age at diagnosis**	74.3 ± 11.0	74.3 ± 11.0	74.6 ± 10.8	73.4 ± 11.2	74 ± 11.1
**eGFR (mL/min/1.73 m^2^)**	64.1 ± 23.3	64.4 ± 22.9	62 ± 25.8 *	59 ± 27.3	48.5 ± 27.2 *^,&,$^
**CKD stage 1 >** **90**	7	6.8	8.7	11.5	6.4
**CKD stage 2** **90–60**	22.6	22.6	24.4	17.3	13.5
**CKD stage 3** **60–30**	18.2	18	20.9	23.7	24.3
**CKD stage 4** **30–15**	3.3	3.1	5.9	7.2	11.4
**Hb at baseline (g/dL)**	12.7 ± 1.9	12.8 ± 1.8	11.4 ± 2.2 *	10.8 ± 2.2 *	11.5 ± 1.9 *
**Hb before event (g/dL)**	12.7 ± 2.0	12.8 ± 1.9	11.4 + 2.2 *	10.2 ± 2.5 *	10.8 ± 2.3 *
**Acute HF hospitalization**	4673 (10.3)	4260 (10.1)	348 (12.0) *	23 (16.5)	42 (12.3)
**Mortality**	10,490 (23.1)	9037 (21.5)	1191 (41.2) *	83 (59.7) *^,&^	179 (52.3) *^,&^
**Comorbidities**					
Anemia	24,479 (53.9)	21,816 (51.9)	2274 (78.6) *	128 (92.1) *^,&^	261 (76.3) *^,$^
Diabetes	22,199 (48.9)	20,501 (48.7)	1456 (50.3)	65 (46.8)	177 (51.8)
Dyslipidemia	27,653 (60.9)	25,732 (61.2)	1642 (56.8) *	78 (56.1)	201 (58.8)
Hypertension	40,663 (89.5)	37,614 (89.4)	2608 (90.2)	121 (87.1)	320 (93.6)
Myocardial infarction	10,087 (22.2)	9178 (21.8)	772 (26.7) *	45 (32.4) *	92 (26.9)
Atrial fibrillation	20,759 (45.7)	18,997 (45.2)	1498 (51.8) *	77 (55.4)	187 (54.7) *
**Treatments**				
Diuretics	36,676 (80.7)	33,817 (80.4)	2452 (84.8) *	118 (84.9)	289 (84.5)
Beta-blockers	22,254 (49.0)	20,526 (48.8)	1489 (51.5) *	66 (47.5)	173 (50.6)
ACEi/ARB	32,415 (71.3)	30,073 (71.5)	2005 (69.3)	84 (60.4) *	253 (74) ^$^
Calcium antagonists	12,906 (28.4)	11,937 (28.4)	818 (28.3)	54 (38.8) *	97 (28.4)
NSAIDs	14,697 (32.3)	13,764 (32.7)	801 (27.7) *	48 (34.5)	84 (24.6) *
Antiplatelets	20,334 (44.8)	18,735 (44.5)	1375 (47.5) *	76 (54.7)	148 (43.3)
Anticoagulants	33,478 (73.7)	30,840 (73.3)	2261 (78.2) *	113 (81.3)	264 (77.2)
Antialdosterone	8579 (18.9)	7869 (18.7)	604 (20.9) *	25 (18)	81 (23.7)

eGFR: estimated glomerular filtration rate; CKD: chronic kidney disease; ACEi/ARB: angiotensin converting enzyme inhibitor/angiotensin receptor antagonists; NSAIDs: non-steroidal anti-inflammatory drugs, (_) percentage; ± standard deviation. * significant differences with the no Hb fall group, and significant differences with the one episode of Hb fall group, ^$^ significant differences with the two or more episodes of Hb fall group. ^&^ significative differences with the on episode of Hb fall group
